# Beyond Visual Assessment of Basal Ganglia Uptake: Can Automated Method and Pineal Body Uptake Assessment Improve Identification of Nigrostriatal Dysfunction on ^18^F-DOPA PET/CT?

**DOI:** 10.3390/ijms24065683

**Published:** 2023-03-16

**Authors:** Shir Hazut Krauthammer, Dan Cohen, Einat Even-Sapir, Hedva Lerman

**Affiliations:** 1 Department of Nuclear Medicine, Tel-Aviv Sourasky Medical Center, 6 Weizmann St., Tel Aviv 6423906, Israel; 2 Sackler Faculty of Medicine, Tel Aviv University, Tel Aviv 6997801, Israel

**Keywords:** ^18^F-DOPA, PET/CT, nigrostriatal dysfunction, Parkinson’s disease, pineal body

## Abstract

The interpretation of ^18^F-DOPA PET/CT performed for assessing nigrostriatal dysfunction (NSD) is usually based on visual assessment of the uptake in the basal ganglia (VA-BG). In the present study, we evaluate the diagnostic performance of an automated method that assesses BG uptake (AM-BG) and of methods that assess pineal body uptake, and examine whether these methods can enhance the diagnostic performance of VA-BG alone. We retrospectively included 112 scans performed in patients with clinically suspected NSD who also had a subsequent final clinical diagnosis provided by a movement disorder specialist (69 NSD and 43 non-NSD patients). All scans were categorized as positive or negative based on (1) VA-BG, (2) AM-BG, and (3) qualitative and semiquantitative assessment of pineal body uptake. VA-BG, AM-BG, assessment of pineal body ^18^F-DOPA uptake by VA (uptake > background), by SUVmax (≥0.72), and by pineal to occipital ratio (POR ≥ 1.57) could all significantly differentiate NSD from non-NSD patients (Pv < 0.01 for all five methods). Of these methods, VA-BG provided the highest sensitivity (88.4%) and accuracy (90.2%). Combining VA-BG with AM-BG did not improve diagnostic accuracy. An interpretation algorithm that combines VA-BG with pineal body uptake assessment by POR calculation increased sensitivity to 98.5%, at the expense of decreased specificity. In conclusion, an automated method that assesses ^18^F-DOPA uptake in the BG and assessment of pineal body ^18^F-DOPA uptake can significantly separate NSD from non-NSD patients, with apparent inferior diagnostic performance when applied alone compared with VA-BG. When VA-BG categorizes a scan as negative or equivocal, assessment of the ^18^F-DOPA uptake in the pineal body has the potential to minimize the rate of false negative reports. Further research is essential to validate this approach and to study the pathophysiologic relationship between ^18^F-DOPA uptake in the pineal body and nigrostriatal dysfunction.

## 1. Introduction

Parkinson’s disease (PD) is a common neurodegenerative disorder characterized by nigrostriatal dysfunction (NSD). PD shares similar clinical signs and symptoms with other neurological disorders, such as Parkinson plus syndromes (PPS, syndromes characterized by NSD as well), secondary parkinsonism (vascular or drug-induced), and essential tremor (ET). While crucial for patient care, treatment selection, and prognosis [1,2], differentiating PD from other etiologies of parkinsonism poses a clinical challenge [3]. Clinical criteria alone fail to prove accurate enough, especially at disease onset, and their accuracy depends greatly on clinical expertise [1]. In recent decades, several single photon emission computed tomography (SPECT) and positron emission tomography (PET) imaging probes were designed, aiming to aid in the clinical management of patients with parkinsonism [4,5].

Presynaptic dopaminergic molecular imaging, using either SPECT (with ^123^I-Ioflupane) or PET (with ^18^F-Fluoro-dihydroxyphenylalanine (^18^F-DOPA)) radioactive tracers, are being commonly employed in clinical practice nowadays. Both techniques show good diagnostic accuracy [6,7], and by identifying NSD they allow one to distinguish PD and PPS from secondary parkinsonism and ET. Beyond the different uptake mechanisms of these radiotracers, ^18^F-DOPA concentrates in cells that express the LAT transporter and the Aromatic L-amino acid decarboxylase (AADC) enzyme, and offers several major advantages over ^123^I-Ioflupane, among which are better image resolution, shorter acquisition time, improved dosimetry, and the lack of potential iodine-induced thyroid-related side effects [8].

Although presynaptic dopaminergic molecular imaging shows great potential, image interpretation of the acquired data lacks an agreed standardization. Interpretation is usually based on visual assessment of the radiotracer uptake pattern in the basal ganglia. Several groups offered semiquantitative methods to aid in image interpretation, such as the striatal to occipital uptake ratio (SOR) calculation [9,10,11], but applying these methods was found to be time consuming and operator dependent [12]. Thus, simple semiquantitative (or automatic) techniques would be of high value if proven to effectively and objectively aid in interpreting imaging data.

While image assessment of ^18^F-DOPA PET/CT imaging focuses on striatal radiotracer uptake, data are scant on extrastriatal changes of dopamine metabolism in patients with PD, particularly in the context of molecular imaging. Only a few studies have reported a considerable increase of ^18^F-DOPA uptake in extrastriatal locations in PD [13,14,15], but its significance remains unclear.

During the past decade, our department gained extensive experience with ^18^F-DOPA PET/CT imaging for identifying NSD. While interpretation was usually based on visual assessment, our impression over time was that extrastriatal ^18^F-DOPA uptake in the pineal body is observed in some but not all patients. We were interested in evaluating the role of pineal body ^18^F-DOPA uptake-based parameters and the role of an automated method that assesses the uptake in the basal ganglia as potential tools to refine the diagnosis of NSD. Specifically, we aim in the current study to: (1) assess the ability of these methods to differentiate NSD and non-NSD patients, using the final diagnosis given to patients by a movement disorder specialist as the gold standard; (2) evaluate the diagnostic performance provided by these methods; and (3) explore whether these methods can be incorporated into the interpretation algorithm of ^18^F-DOPA PET/CT and thus refine NSD diagnosis.

## 2. Results

### 2.1. Evaluation of Basal Ganglia ^18^F-DOPA Uptake by Visual Assessment and Automated Method to Identify NSD

Among the total 112 study patients, 69 (61.6%) of whom were diagnosed as having NSD according to the movement disorder specialist, 64/112 (57.1%) and 40/112 (35.7%) were categorized as positive scans by visual assessment of the uptake in the basal ganglia (VA-BG) and by an automated method that assesses the uptake in the basal ganglia (AM-BG), respectively.

Positivity on VA-BG significantly differentiated NSD patients from non-NSD patients (61/69 vs. 3/43, 88.4% vs. 7.0%, Pv < 0.01, Table 1). Positivity on AM-BG significantly differentiated NSD patients from non-NSD patients as well (37/69 vs. 3/43, 53.6% vs. 7.0%, Pv < 0.01, Table 1).

Table 2 summarizes the diagnostic performance parameters calculated for VA-BG and AM-BG. While both methods showed the same specificity, AM-BG resulted in lower sensitivity, accuracy, and NPV. Combining these two methods could only minorly improve the sensitivity and NPV provided by VA-BG alone.

### 2.2. Evaluation of Pineal Body ^18^F-DOPA Uptake Parameters to Identify NSD

**Figure 1:**
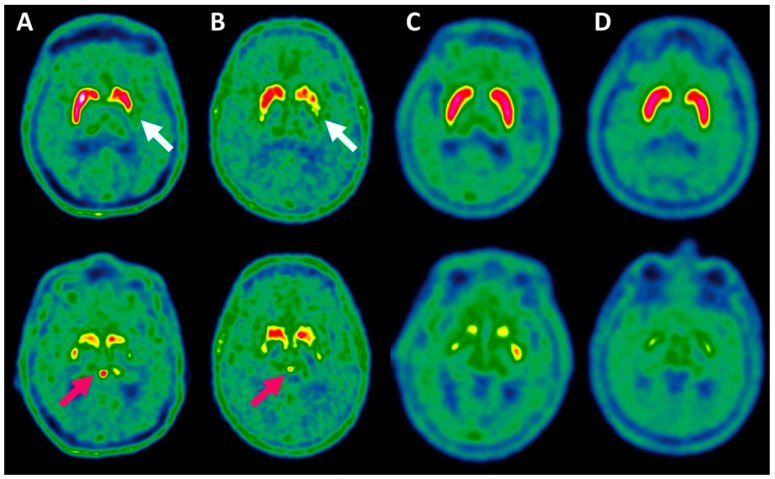
Visual assessment of ^18^F-DOPA uptake in the basal ganglia (upper row) and in the pineal body (lower row) in patients with nigrostriatal dysfunction (NSD) (**A**,**B**) and in patients with non-NSD conditions (**C**,**D**). In NSD patients, notice the asymmetric decreased uptake pattern in the basal ganglia (white arrows) with concurrent increased pineal body uptake compared to the background (pink arrows). Non-NSD patients demonstrated normal ^18^F-DOPA uptake pattern in the basal ganglia with virtually absent uptake in the pineal body.

Among the total 112 study patients, 69 (61.6%) of whom were diagnosed as having NSD according to the movement disorder specialist, 65/112 (58.0%) had pineal body ^18^F-DOPA uptake higher than the background (see two illustrative cases in Figure 1). Applying this criterion, positivity significantly differentiated NSD patients from non-NSD patients (48/69 vs. 17/43, 69.6% vs. 39.5%, Pv < 0.01, Table 3).

**Figure 2:**
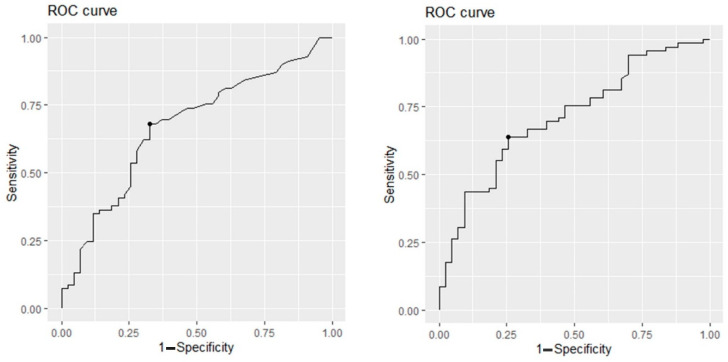
Receiver operating characteristic (ROC) curves for NSD identification according to pineal body SUVmax (**left**, AUC = 0.67) and pineal to occipital cortex uptake ratio (POR, **right**, AUC = 0.71).

Plotting the sensitivity against the (1-specificity) in ROC curves for the maximum standardized uptake value (SUVmax) and for the pineal to occipital cortex uptake ratio (POR) (Figure 2), the calculated AUCs were 0.67 and 0.71, respectively. The optimal cutoff values that maximize the sum of sensitivity and specificity for each variable were identified as 0.72 and 1.57 for SUVmax and POR, respectively. Applying these cutoff values, both binary criteria significantly differentiated NSD patients from non-NSD patients (Table 3).

Table 4 summarizes the diagnostic performance parameters calculated for VA-BG and for the binary qualitative and semiquantitative-based criteria used to assess ^18^F-DOPA uptake in the pineal body. Among these criteria, POR ≥ 1.57 showed the highest specificity (74.4%), accuracy (67.8%), and PPV (80%), with only slightly lower sensitivity, and NPV.

In order to evaluate the potential application of pineal body uptake parameters to refine the diagnostic performance of VA-BG alone, we evaluated whether positivity in at least one of these methods or positivity in both criteria result in improved diagnostic performance (Table 4). We found that positivity by at least one criterion (VA-BG or POR ≥ 1.57) resulted in improved sensitivity (98.5%) while only moderately lowering the specificity provided by each criterion alone.

## 3. Discussion

The diagnosis of NSD, and particularly of PD, mostly relies on clinical criteria, which undergo continual adjustments [16]. Molecular imaging has been shown to have high accuracy and to assist in making a diagnosis of NSD [17,18]. Although dictated by general guidelines [19], the interpretation of ^18^F-DOPA PET/CT lacks uniformity and depends greatly on interpreting physicians’ expertise. The purpose of this single-center retrospective study was to evaluate the diagnostic ability of different methods in discriminating between patients with NSD and patients without NSD, using the final clinical diagnosis provided by a movement disorder specialist as the gold standard. More specifically, the current study evaluated whether using an automated method that assesses BG ^18^F-DOPA uptake or pineal body ^18^F-DOPA uptake parameters could refine the diagnostic performance of VA-BG alone.

Comparing the diagnostic performance of all of the studied criteria, our results suggest that visual assessment of the ^18^F-DOPA uptake pattern in the basal ganglia was superior to all other methods, reaching an accuracy of more than 90%. It should be noted, however, that given that this criterion was the only one reported to the movement disorder specialist, together with the role of the ^18^F-DOPA PET/CT report in making the clinical diagnosis of NSD, an inherent inevitable bias could partially explain the high diagnostic performance parameters provided by this criterion. As the diagnosis of NSD lacks neuropathological confirmation in living patients, the final clinical diagnosis provided by the movement disorder specialist is considered acceptable in similar studies, which also discussed this aforementioned pervasive limitation [8,9,20,21].

Regarding the AM-BG evaluated in our study, even though this automated method could significantly differentiate NSD from non-NSD patients in our cohort, it showed inferior performance compared to VA-BG, with sensitivity and accuracy reaching 53.6% and 68.8% only. While the DaTQUANT software was developed and optimized to assess ^123^I-FP-CIT SPECT images, the modified version applied in the current study enabled loading ^18^F-DOPA PET images, but neither the template, automatic VOI generation, nor any other component were optimized for analyzing ^18^F-DOPA PET images, which differ from ^123^I-FP-CIT SPECT images. Other studies that examined other automated methods, mainly based on automated SOR calculation, revealed better results [12,22]. In a study by Arena et al. on 60 patients, the authors reported the same level of discrimination between NSD and non-NSD patients applying manual and automated methods [22]. Chung et al. showed no significant difference between SOR values obtained using their suggested automated method and a manual analysis in 21 patients with PD and six healthy controls [12].

One of the motivations we had to perform this study was to assess the pineal body ^18^F-DOPA uptake as a tool to differentiate patients with NSD from patients with non-NSD pathologies. Through years of experience, we had the impression that higher pineal body ^18^F-DOPA uptake correlated with a pathologic pattern of ^18^F-DOPA uptake in the basal ganglia. Before performing the current study, this observation lacked profound scientific validation or physiologic explanation. Our results demonstrate that simple qualitative visual assessment of ^18^F-DOPA pineal body uptake, as well as semiquantitative parameters, significantly distinguished patients with NSD from those without NSD. Moreover, our findings provide the community of interpreting physicians with validated cutoffs of SUVmax and POR that can be incorporated into routine clinical work.

Given these intriguing results regarding the potential role of pineal body ^18^F-DOPA uptake, we reviewed relevant literature aiming to better understand the roots of this observation. While the decrease in dopamine metabolism and ^18^F-DOPA uptake in the basal ganglia in NSD has been extensively discussed [23,24], extrastriatal dopamine metabolism has been rarely studied. Pineal body cells, most commonly known for their role in the rhythmic secretion of melatonin, express AADC, an enzyme that catalyzes several different reactions, including L-DOPA decarboxylation to dopamine and 5-hydroxytryptophan decarboxylation to serotonin (the precursor for melatonin) [25]. Some studies that evaluated pathologies with decreased dopaminergic neuron mass (including PD) in fact reported an increase in AADC activity or synthesis [26,27], with the main hypothesis proposed to explain this phenomenon being a compensatory extrastriatal enzyme upregulation [13,14]. On molecular imaging, only a few previous studies have reported the relationship between pineal body ^18^F-DOPA uptake and NSD. Gehaemi et al. reported that in 21 patients with PD, ^18^F-DOPA uptake in the pineal body was higher compared to healthy subjects [13]. Moore et al. reported that this increased uptake was specifically related to patients with early PD [14].

**Figure 3:**
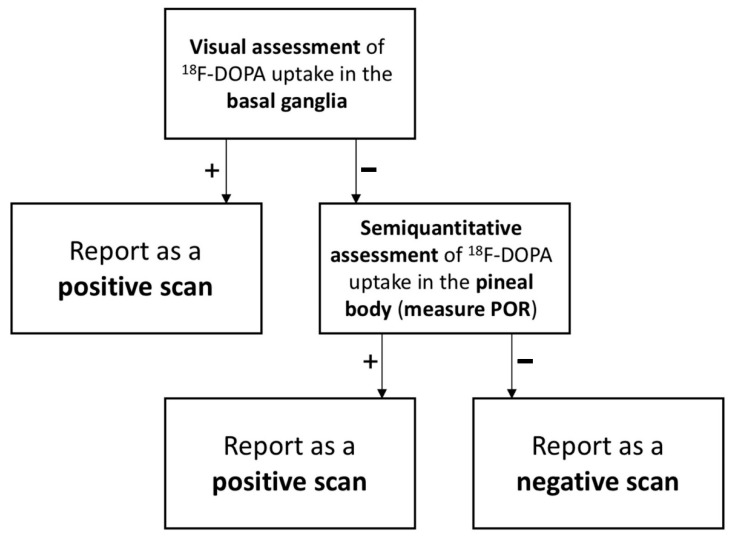
Our proposed algorithm for nigrostriatal dysfunction identification on ^18^F-DOPA PET/CT.

The results of the current study suggest that incorporating the assessment of ^18^F-DOPA uptake in the pineal body can enhance the diagnostic accuracy of ^18^F-DOPA PET/CT interpretation. In Figure 3, we propose a novel and simple-to-use approach to ^18^F-DOPA PET/CT study interpretation. In cases when a scan is categorized as positive based on visual assessment of basal ganglia ^18^F-DOPA uptake, further evaluation of pineal body uptake is not necessary. However, when a scan appears to be negative or equivocal based on VA-BG, we suggest assessing POR and including this value in the final report. Interpreting physicians may consider a scan to be positive if POR is equal to or greater than 1.57. Moore et al.’s observation that pineal body uptake is associated with early stage pathology [14] may indicate that adopting the adjusted algorithm we propose (Figure 3) has the potential to increase the sensitivity of ^18^F-DOPA PET/CT in diagnosis of early NSD. In our cohort, retrospective application of this algorithm resulted in lowering the false negative rate from 7.1% to 0.9% and improving sensitivity to as high as 98.5%.

The present study has several limitations. First, as discussed above, the use of the final diagnosis provided by movement disorder specialists as gold standard is suboptimal (compared with an objective histopathological confirmation) but pervasive in studying this group of patients. Second, although the number of cases included in this work is higher than the number included in previous similar studies, validating our results and the suggested algorithm on other cohorts is imperative. Third, further research is essential to better study the pathophysiologic relationship between ^18^F-DOPA uptake in the pineal body and nigrostriatal dysfunction. 

## 4. Materials and Methods

We reviewed all cases of patients who underwent an ^18^F-DOPA PET/CT brain scan in the nuclear medicine department at Tel-Aviv Sourasky Medical Center between February 2015 and December 2021, and included in the current study all cases that met the following three inclusion criteria: (1) patients were referred to imaging due to clinical parkinsonism of uncertain origin; (2) the final clinical diagnosis of NSD versus other causes of parkinsonism given to the patient by a movement disorder specialist was available on their medical records; (3) application of the studied automated method was available for the scan.

### 4.1. Patient Population

A total of 112 cases met the inclusion criteria and were included in the current study. Median age of the included patients was 68 (IQR, 62.5–68), 70/112 (62.5%) were male patients. The eventual diagnosis as it was documented by the movement disorder specialist was NSD in 69 patients (62 patients diagnosed with PD and 7 patients diagnosed with PPS), and non-NSD conditions in 43 patients (21 patients diagnosed with ET, 9 patients with secondary parkinsonism, mainly drug induced, and the remainder were diagnosed with other neurologic conditions such as normal pressure hydrocephalus).

### 4.2. PET/CT Acquisition

The patients were instructed to fast for 4 h prior to ^18^F-DOPA administration. Medications that could interfere with ^18^F-DOPA uptake were held, and the patients were interviewed prior to the scan to verify the changes in their medication schedule. Patients received 200–400 MBq of ^18^F-DOPA intravenously approximately 70 min prior to the scan. The studies were performed on PET/CT scanners (GE Healthcare; DISCOVERY 690 and DISCOVERY MI) in a 3-dimensional scanning mode, according to our standard protocol [19].

### 4.3. Investigated Imaging Criteria

We applied the following imaging interpretation methods in all of the included cases: visual assessment of the uptake in the basal ganglia (VA-BG), an automated method to assess the uptake in the basal ganglia (AM-BG), and pineal body uptake assessment. We recorded the binary conclusion (positive versus negative for NSD) drawn by each method in all cases.

**Visual assessment of ^18^F-DOPA uptake in the basal ganglia (VA-BG).** All scans were assessed by a nuclear medicine physician (HL) who has decades of experience in reading dopaminergic nuclear imaging. A scan was considered positive for NSD when VA revealed a relative decrease in ^18^F-DOPA uptake in the basal ganglia (unilateral or bilateral) in a typical pattern (i.e., asymmetric reduction of ^18^F-DOPA uptake in the distal rather than the proximal putamen) [28].

**Figure 4:**
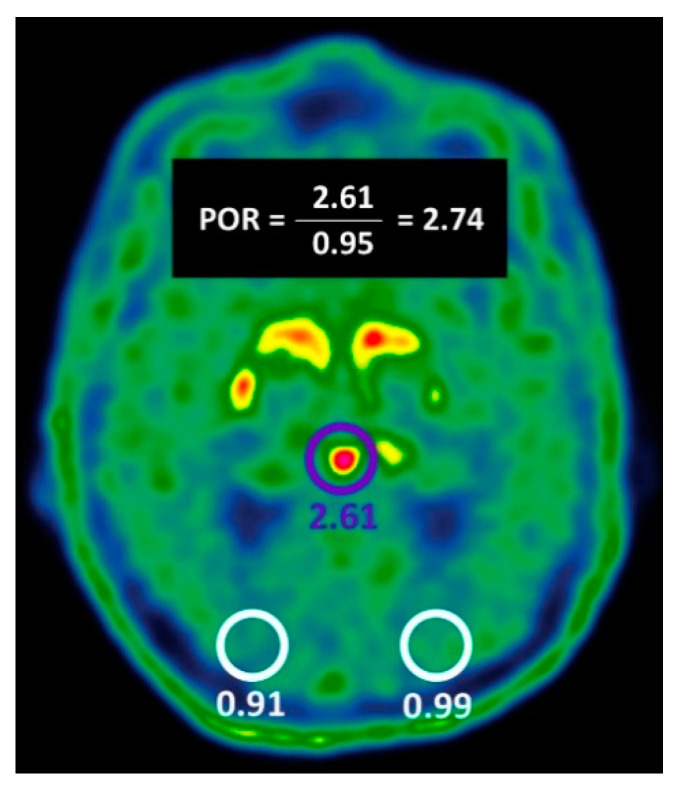
Illustration of POR calculation. VOIs are drawn on the pineal body and the right and left occipital cortices. The SUVmax in the pineal body VOI (purple), and the SUVmean values in the occipital cortices VOIs (white) are recorded. Pineal to occipital cortex uptake ratio (POR) is calculated as the ratio between the SUVmax value in the pineal body and the average SUVmean values in the occipital cortex. Based on the present study, POR value ≥ 1.57 was associated with the diagnosis of nigrostriatal dysfunction.

**Pineal body uptake assessment.** In all scans, we visually assessed and recorded whether the pineal body uptake exceeded the uptake observed in the adjacent background (Figure 1). Furthermore, the maximum standardized uptake value (SUVmax) in the pineal body, and the mean standardized uptake values in the right and left occipital cortices (RT-SUVmean and LT-SUVmean, respectively) were measured. The ratio between pineal body SUVmax and the average of RT-SUVmean and LT-SUVmean were recorded in all scans as pineal to occipital cortex uptake ratio (POR, see illustration of POR calculation in Figure 4). SUV values were automatically calculated by means of 1.5 cm^3^ spherical VOIs.

**Automated method.** We used a modified version of the commercially available DaTQUANT software (GE Healthcare) [29]. DaTQUANT is approved for automated semi-quantitative analysis of ^123^I-FP-CIT. Briefly, the methods used in DaTQUANT are as follows: the quantification method is based on fully automated registration of the patient’s scan to a predefined template [30], followed by interrogation of the uptake in the striatal regions using a volume of interest (VOI) atlas. The atlas defines VOIs corresponding to the following regions: caudate, anterior putamen, posterior putamen, striatum, and background. The image registration in DaTQUANT, and in the modified DaTQUANT which was applied in the current study for AM-BG, uses a DaTscan and ^18^F-DOPA templates which were created from a set of normal subjects. The applications perform automatic non-rigid registration of each subject’s imaging input to the mentioned template. Once the registration has been completed, the VOIs are applied to the data and are used for calculation of the striatal binding ratio (SBR) [31], calculated as the ratio between striatal counts minus background counts and background counts. The software compares the calculated SBR of a given patient to the normal reference values in terms of standard deviations (Z-scores), and an abnormal result is defined via a cutoff of >2SD from reference values.

### 4.4. Statistical Analysis

Categorical data were described with contingency tables that included frequency and percent. Continuous variables were evaluated for normal distribution and reported as median and interquartile range (IQR). To compare rates of categorical variables (e.g., to compare positivity rates among NSD and non-NSD patients in order to assess the ability of the studied methods to differentiate between them), Pearson’s χ^2^ test and Fisher’s exact test were used. For each of the binary studied methods, the diagnostic performance parameters (sensitivity, specificity, accuracy, positive predictive value (PPV), and negative predictive value (NPV)) were calculated. The continuous variables (pineal body SUVmax and POR) were analyzed applying the “cutpointr” R package [32], which was used to plot receiver operating characteristic (ROC) curves, calculate the area under the curves (AUC), and identify the optimal cutoff values that maximize the sum of sensitivity and specificity for each variable. To assess the diagnostic performance of these continuous variables as binary, they were dichotomized at the optimal cutoff values. A two-sided *p* value of <0.05 was considered statistically significant. SPSS software (IBM SPSS Statistics for Windows, version 27, IBM Corp., Armonk, NY, USA, 2017) and the open-source statistics software R (version 4.0.5, R Foundation for Statistical Computing, Vienna, Asutria) were used for statistical analysis.

## Figures and Tables

**Table 1:** Differentiation between NSD and non-NSD patients based on visual assessment and automated method-based assessment of ^18^F-DOPA uptake in the basal ganglia.

	Positivity among NSD Patients	Positivity among Non-NSD Patients	*p* Value
VA-BG	61/69 (88.4%)	3/43 (7%)	<0.01
AM-BG	37/69 (53.6%)	3/43 (7%)	<0.01

NSD, nigrostriatal dysfunction; VA-BG, visual assessment of ^18^F-DOPA uptake in basal ganglia; AM-BG, automated method that assesses ^18^F-DOPA uptake in basal ganglia.

**Table 2:** Diagnostic performance parameters provided by visual assessment and automated method-based assessment of ^18^F-DOPA uptake in the basal ganglia.

	Sensitivity	Specificity	Accuracy	PPV	NPV
VA-BG	61/69 (88.4%)	40/43 (93.0%)	101/112 (90.2%)	61/64 (95.3%)	40/48 (83.3%)
AM-BG	37/69 (53.6%)	40/43 (93.0%)	77/112 (68.8%)	37/40 (92.5%)	40/72 (55.6%)
VA-BG **OR** AM-BG	63/69 (91.3%)	38/43 (88.4%)	101/112 (90.2%)	63/68 (92.6%)	38/44 (86.4%)
VA-BG **AND** AM-BG	35/69 (50.7%)	42/43 (97.7%)	77/112 (68.8%)	35/36 (97.2%)	42/76 (55.3%)

VA-BG, visual assessment of ^18^F-DOPA uptake in basal ganglia; AM-BG, automated method that assesses ^18^F-DOPA uptake in basal ganglia; PPV, positive predictive value; NPV, negative predictive value.

**Table 3:** Differentiation between NSD and non-NSD patients based on pineal body ^18^F-DOPA uptake assessment.

	Positivity among NSD Patients	Positivity among Non-NSD Patients	*p* Value
VA-PB	48/69 (69.6%)	17/43 (39.5%)	<0.01
SUVmax ≥ 0.72	47/69 (68.1%)	14/43 (32.6%)	<0.01
POR ≥ 1.57	44/69 (63.8%)	11/43 (25.6%)	<0.01

NSD, nigrostriatal dysfunction; VA-PB, visual assessment of ^18^F-DOPA uptake in pineal body; POR, pineal to occipital cortex uptake ratio.

**Table 4:** Diagnostic performance parameters provided by pineal body ^18^F-DOPA uptake assessment.

	Sensitivity	Specificity	Accuracy	PPV	NPV
VA-BG	61/69 (88.4%)	40/43 (93.0%)	101/112 (90.2%)	61/64 (95.3%)	40/48 (83.3%)
VA-PB	48/69 (69%)	26/43 (60%)	74/112 (66%)	48/65 (74%)	26/47 (55%)
SUVmax ≥ 0.72	47/69 (68%)	29/43 (67.4%)	76/112 (67.8%)	47/61 (77.0%)	29/51 (56.8%)
POR ≥ 1.57	44/69 (63.7%)	32/43 (74.4%)	76/112 (67.8%)	44/55 (80%)	32/57 (56.1%)
VA-BG **OR** POR ≥ 1.57	68/69 (98.5%)	29/43 (67.4%)	97/112 (86.6%)	68/82 (82.9%)	29/30 (96.6%)
VA-BG **AND** POR ≥ 1.57	37/69 (53.6%)	43/43 (100%)	80/112 (71.4%)	37/37 (100%)	43/75 (57.3%)

VA-BG, visual assessment of ^18^F-DOPA uptake in basal ganglia; VA-PB, visual assessment of ^18^F-DOPA uptake in pineal body; POR, pineal to occipital cortex uptake ratio; PPV, positive predictive value; NPV, negative predictive value.

## Data Availability

The datasets used and/or analyzed during the current study are available from the corresponding author on reasonable request.

## Abbreviations

^18^F-DOPA: ^18^F-Fluoro-dihydroxyphenylalanine; PET/CT: Positron emission tomography/computed tomography; NSD: Nigrostriatal dysfunction; PD: Parkinson’s disease; PPS: Parkinson plus syndromes; VA-BG: Visual assessment—basal ganglia; AM-BG: Automated method—basal ganglia; VA-PB: Visual assessment—pineal body; SUV: Standardized uptake value; POR: Pineal to occipital cortex uptake ratio; AADC: Aromatic L-amino acid decarboxylase; PPV: positive predictive value; NPV: negative predictive value.
